# Validity of Italian version of the Child Perceptions Questionnaire (CPQ_11-14_)

**DOI:** 10.1186/1472-6831-13-55

**Published:** 2013-10-16

**Authors:** Armando Olivieri, Roberto Ferro, Luca Benacchio, Alberto Besostri, Edoardo Stellini

**Affiliations:** 1 Physician epidemiologist, Epidemiology Unit Prevention Department - Local Health Unit 15 Veneto Region, via Cao del Mondo, 35012 Camposampiero PD, Italy; 2 Dentist, Dentistry Unit Cittadella Hospital - Local Health Unit 15 Veneto Region, via Casa di Ricovero 40, 35013 Cittadella PD, Italy; 3 Statistician, Epidemiology Unit Prevention Department - Local Health Unit 15 Veneto Region, via Cao del Mondo, 35012 Camposampiero PD, Italy; 4 Dental Institute University of Padua - Clinica Odontoiatrica, Via Venezia, 90, 35131 Padova, Italy

**Keywords:** Oral health, Quality of life, CPQ, Validity, Caries, Social position

## Abstract

**Background:**

The Child Perceptions Questionnaire (CPQ_11-14_) is the most commonly used indicator of child oral health-related quality of life (OHRQoL), and its validity and reliability have been studied both in English and in other linguistic contexts. The aim of this study was to develop a CPQ_11-14_ for use in Italy and to test its validity in a random sample of fourteen year-old Italian adolescents.

**Methods:**

Once the CPQ_11-14_was translated into Italian and adapted for an Italian public, five hundred sixty-one adolescents were recruited for testing. Parents rated their social status; the children/adolescents were administered the questionnaire and underwent a dental examination during which their dental status was taken and recorded. Cronbach's alpha was used to assess the questionnaire’s internal consistency. Spearman's correlation coefficients were calculated to assess construct validity between the total and subscale scores and the respondents’ global ratings on oral health and well-being. Discriminant validity was analysed using the Kruskal-Wallis or Mann–Whitney tests in groups defined by gender, social position, caries experience and previous or no orthodontic treatment.

**Results:**

The mean score on the CPQ_11-14_ was 15.4 (SD=11.9), and the scores on all the domains were found to be highly skewed. Cronbach's alpha ranged from 0.85 to 0.90. The global ratings on oral health and well-being were correlated to the total score and to the sub-scores except for those regarding the functional limitations. There were significant differences in the two genders, in the groups that had already or had not yet undergone orthodontic treatment, and in the social classification groups, while the difference between those who had and those who did not have caries experience did not reach statistical significance.

**Conclusions:**

The Italian version of the CPQ_11-14_ appears to be a reliable, valid instrument for Italian children/adolescents.

## Background

Quality of life (QoL) is a broad multidimensional concept that usually includes subjective evaluations of both positive and negative aspects of life. On an individual level, the concept of health-related quality of life (HRQoL) includes physical and mental health perception and correlates such as health risks and conditions, functional status, social support, and socioeconomic status [1].

There can be no doubt that oral health can significantly affect HRQoL [2], thus oral health-related quality of life (OHRQoL) is an important aspect of a more complex state of being. OHRQoL has been widely studied over the past two decades and many tools have been developed, mostly for adults, aiming to assess not only physical well-being but also functional, psychological, and social satisfaction in relation to oral health [3-7].

Increasing interest has been dedicated to OHRQoL in children. A systematic review [8] identified three validated OHRQoL instruments designed to assess the impact of oral conditions on quality of life in children and adolescents: Child-Oral Impacts of Daily Performances index (Child-OIDP) [9], Child Oral Health Impact Profile (COHIP) [10] and Child Perceptions Questionnaire (CPQ) [11].

The CPQ_11-14_ is, nevertheless, the most commonly used instrument and its validity and reliability have been studied in English-speaking children/adolescents in countries such as Canada, the United Kingdom, New Zealand, and Australia [11-14]. The questionnaire has also been translated and validated in other cultural and linguistic contexts such as those in Arabia, Uganda, Brazil, Portugal, China and Denmark [15-19]. Despite its widespread diffusion worldwide, the CPQ_11-14_ has never been adapted for use as an epidemiological tool in Italy. In order to assess the occurrence of malocclusion traits and the related treatment need it is important to evaluate individuals’ perception about their oral health, besides traditional clinical indicators. Not all patients with malocclusions report concerns about their appearance or about how malocclusions impact on functional well-being [20,21].

As a consequence we planned a study on malocclusion prevalence in a sample of 14-years-old adolescents, collecting clinical data on oral health status and treatment need as well as self perception of OHRQoL. The aim of this present study was, therefore, to develop an Italian version of the CPQ_11-14_ and to assess the instrument’s validity in an Italian population of adolescents.

## Methods

This study was part of a research project aiming to evaluate the occurrence of malocclusion in permanent dentition in a sample of 14 year-old Italian adolescents. Approved by the Local Ethics Committee of Padua, the cross-sectional survey using random cluster sampling was carried out between October 2007 and May 2008. The area being sampled was made up of 28 municipalities (Health District n.15 – where 240,000 inhabitants resided) located in the centre of the Veneto region (Northeast Italy). The population being studied was made up of children/adolescents attending the last year of middle school - thus fourteen-year-olds. Inclusion criteria were, in fact, year of birth (1994) and consent forms signed by parents.

School authorities were contacted by members of the research group and fully informed about all aspects of the study. Teachers were asked to send home a description of the study explaining its methodology and purpose and asking for parents’ collaboration. Those parents intending to give permission for their children/adolescents to participate were asked to complete a self-report questionnaire about their social position (occupational status), to sign a consent form, and to send both back to school. In accordance with Caiazzo et al.’ s work [22], occupational levels were classified into 4 classes: high class (managers, professionals), clerks (clerical employees, managerial and technical occupations), self-employed (owners of small companies and artisans), and working class (manual workers, skilled and unskilled, housewives).

The sample size was calculated going on the assumption that the prevalence of malocclusion in adolescents is about 50%. Using an interclass coefficient of 0.5, the estimated number of children/adolescents was set at 1100. The mean number of children/adolescents in each class was approximately 22; each class was identified as a cluster. There were 107 third year of middle school classes in the area studied and 51 of these were randomly chosen. The theoretical number of the children/adolescents that could be enrolled in the study was therefore 1187. Of these, 295 (24.86%) did not return consent forms signed by their parents to school and were thus excluded from the study. Another 110 were excluded because they were not born in 1994. One hundred and fifteen were excluded because they were absent on the day the dental examination was carried out and 106 declined to participate. In the end, 561 adolescents (269 females = 48 % + 292 males = 52%) were recruited.

Oral health-related quality of life was measured using the CPQ_11-14_ which consists of 37 items distributed over 4 domains (oral symptoms, functional limitation, emotional well-being and social well-being) investigating the frequency of events related to oral health over the previous three months’ time [11]. Response options and scores were: 'Never’ (scoring 0); 'Once or twice’ (1); 'Sometimes’ (2); 'Often’ (3); and 'Every day or almost every day’ (4). The final score, computed by summing the scores on all the items, ran from 0 to 148. Higher scores indicate higher impact of oral conditions on quality of life. The questionnaire [11] also included two direct questions asking respondents to give a global rating of their oral health and the extent to which it affected their overall well-being. The questions were worded in the following way: “Would you say that the health of your teeth, lips, jaws and mouth is…?” and “How much does the condition of your teeth, lips, jaws or mouth affect your life overall?” The responses were scored in the following way: with regard to a global rating of oral health: (0) excellent, (1) very good, (2) good, (3) fair and (4) poor; with regard to overall well-being: (0) not at all, (1) very little, (2) somewhat, (3) a lot and (4) very much.

The English CPQ version was translated into Italian using the forward-backward technique following the approach outlined in the literature [23-26]. The aim was to produce a questionnaire in Italian whose meaning matched as perfectly as possible the English original. The initial translation was carried out by two Italian dentists fluent in both Italian and English. The Italian draft that was produced was given to a mother-tongue English consultant fluent in Italian (not a member of our team and who had never had access to the original version) who was instructed to translate the Italian text back into English. The two English versions (the original and the one produced by translating it back into English) were found to be semantically similar and only minor adjustments needed to be made to the Italian version.

Italian version of the questionnaire was utilized by us to carry out our survey in an Italian adolescent population. All of the adolescents recruited were administered the CPQ_11-14_ and underwent a dental examination. The clinical assessments were carried out by one of two qualified dentists (RF and AB) under standardized conditions and optimal artificial lighting, using air drying, a plain mirror, and a WHO-CPI probe. Oral health status and caries experience were assessed (Decayed/Missing/Filled Surface=dmfs/DMFS). Carious lesions were diagnosed when there were cavities at dentinal level D_3_. Bitewing radiographs were not used for caries diagnosis. The presence of any orthodontic appliance was recorded.

### Analysis

The CPQ_11-14_ score was computed by summing the global score of all 37 items; and the scores for each of the four domains (sub-scales) were also calculated. The four sub-scales were thus divided: oral symptoms (6 items), functional symptoms (9 items), emotional well-being (9 items), and social well-being symptoms (13 items).

As the distribution of the scores was non-normal, besides using the mean and the standard deviation of the data, they were described using the median and the interquartile range. Cronbach’s alpha was used to estimate the questionnaire’s internal consistency. For logistical and organizational reasons test-retest reliability of the CPQ_11-14_ was not assessed. As organizing another dental examination session at all of the schools participating in our study would have been a complex endeavour, the feasibility of a retest appeared problematic. As a consequence no test-retest of the questionnaire was undertaken.

Associations between the scores on each domain and the respondents’ global rating of oral health and overall well-being were analyzed using Spearman’s correlation coefficient to test the questionnaire’s construct validity.

Discriminant validity was tested by comparing the average scores between groups defined by gender, social position, caries experience and orthodontic treatment already experienced: as the scores were not normally distributed the statistical significance of differences between groups was determined using the Kruskal-Wallis or Mann–Whitney tests. Data were analysed using Stata rel. 11.2 (Stata Corporation, College Station, TX, USA).

## Results

**Figure 1:**
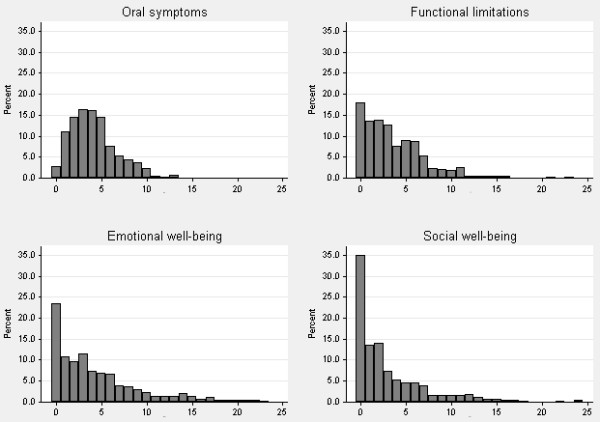
Distribution of scores on the CPQ11-14 sub-scales.

Out of the 561 (52% males) adolescents recruited, 37% were caries free and 21% had already undergone orthodontic treatment. Scores ranged between 0 and 81 and the mean score was 15.4 (SD=11.9) (Table 1). The response rate to the items on the questionnaire was 98%: the section with the highest number (8) of blanks was the functional limitations domain. There were, in fact, 8 items left blank in the functional limitations domain, 6 items in the emotional well-being and 4 items in the social well-being. There were no unanswered items in the oral symptoms section. No child missed more than one item. Scores were found to be highly skewed in all the domains (Figure 1).

**Table 1:** Baseline characteristics of the population studied

**CPQ scores**											
	**number**	**Oral symptoms**		**Functional limitations**		**Emotional well-being**		**Social well-being**		**Overall**	
		**mean (sd)**	**median**	**mean (sd)**	**median**	**mean (sd)**	**median**	**mean (sd)**	**median**	**mean (sd)**	**median**
*gender*											
boys	292	3.9 (2.4)	4	3.3 (3.0)	3	3.6 (3.6)	3	2.8 (3.8)	1	13.5 (9.9)	12
girls	269	4.4 (2.7)	4	4.1 (3.8)	3	5.5 (5.7)	4	3.6 (4.6)	2	17.5 (13.6)	15
*social position*											
high class	57	3.7 (2.2)	3	2.9 (2.8)	2	3.6 (4.2)	2	2.5 (3.7)	1	12.3 (10.2)	9
clerks	178	4.4 (2.5)	4	3.7 (3.4)	3	4.4 (4.8)	3	2.8 (3.8)	2	15.2 (11.2)	13
self employed	117	4.0 (2.5)	4	3.3 (3.1)	2	4.1 (5.1)	3	2.7 (3.5)	1	14.1 (10.8)	12
working class	187	4.2 (2.7)	4	4.3 (4.0)	3	5.2 (5.0)	4	4 (5.0)	2	17.7 (13.6)	15
n.r.	22	4.1 (2.4)	4	2.7 (2.6)	2	3.1 (3.1)	2	2.8 (4)	1	12.8 (9.5)	11
caries experience (DMFS)*											
0	205	4.4 (2.5)	4	3.7 (3.2)	3	4.2 (4.6)	3	2.9 (3.5)	2	15.0 (10.4)	13
1-2	110	3.8 (2.4)	3.5	3.5 (3.6)	2	4.5 (4.9)	3	3.3 (4.4)	2	14.9 (12.6)	11
3-5	131	4.2 (2.5)	4	3.9 (3.6)	3	4.3 (4.7)	3	3.2 (4.5)	2	15.6 (12.5)	13
>=6	113	4.2 (2.6)	4	3.8 (3.6)	3	5.3 (5.2)	4	3.3 (4.9)	2	16.8 (13.4)	14
had already undergone orthodontic treatment*											
no	444	4.0 (2.5)	4	3.3 (3.2)	2.5	4.4 (4.7)	3	2.8 (4.1)	1	14.5 (11.5)	12
yes	115	4.8 (2.7)	4	5.2 (4.0)	4	4.7 (5.2)	3	4.4 (4.5)	3	19.0 (13.0)	16

*two boys were not at school the day the children underwent the dental examination.

### Internal consistency

Cronbach’s alpha resulted 0.88 for the total score and ranged from 0.85 for social well-being to 0.90 for oral symptoms, indicating an acceptable to good internal consistency (Table 2).

**Table 2:** **Internal consistency of the CPQ**_**11-14 **_**scores**

	**number of items**	**Cronbach's alpha (n=561)**
Total scale	37	0.88
*Subscales*		
Oral symptoms	6	0.90
Functional limitations	9	0.86
Emotional well-being	9	0.86
Social well-being	13	0.85

### Construct validity

The correlations between the global ratings on oral health and overall well-being and the total score were found to be highly significant (Table 3). The correlations between the global ratings and the domains were all significant with the exception of the correlation between oral health and functional limitations.

**Table 3:** Construct validity: Total and sub-scale score correlations with global ratings of oral health and well-being

		**Global rating**			
	**mean CPQ scores (sd)**	**oral health**		**oral well-being**	
		r*	**p-value**	r*	**p-value**
Total scale	15.4 (11.9)	0.1828	<0.0001	0.1898	<0.0001
*Subscales*					
Oral symptoms	4.2 (2.5)	0.0886	0.0362	0.0975	0.0211
Functional limitations	3.7 (3.5)	0.0598	0.1573	0.0911	0.0313
Emotional well-being	4.4 (4.8)	0.2163	<0.0001	0.2123	<0.0001
Social well-being	3.1 (4.2)	0.1772	<0.0001	0.1511	0.0003

*Spearman's correlation coefficient.

### Discriminant validity

There were significant gender differences in the total as well as in each sub-scale score (Table 4). The children/adolescents who had already undergone orthodontic treatment produced significantly higher scores in functional limitations and social well-being domains as well as in the total score compared to those who had not. The respondents with caries experience did not have statistically significant higher scores on any of the subscale or total scores. There was a significant gradient within the social classes overall as well as in the emotional and social well-being sub-scale scores.

**Table 4:** **Discriminant validity: CPQ**_**11-14 **_**scores according to sex, social position, caries experience and orthodontic treatment**

	**number**	**Oral symptoms**	**Functional limitations**	**Emotional well-being**	**Social well-being**	**Total score**
		**median (IQR)**	**median (IQR)**	**median (IQR)**	**median (IQR)**	**median (IQR)**
*GENDER*						
boys	292	4 (3)	3 (4)	3 (5)	1 (4)	12 (13)
girls	269	4 (4)	3 (5)	4 (7)	2 (5)	15 (16)
Wilcoxon rank-sum test		p= 0.0212	p= 0.0352	p= 0.0009	p= 0.0450	p= 0.0007
*SOCIAL POSITION*						
high class	57	3 (3)	2 (4)	2 (6)	1 (4)	9 (12)
clerks	178	4 (3)	3 (4)	3 (5)	1.5 (4)	13 (14)
self employed	117	4 (3)	2 (4)	2 (6)	1 (3)	12 (13)
working class	187	4 (4)	3 (5)	4 (7)	2 (6)	15 (15)
n.r.	22	4 (3)	2 (5)	2 (5)	1 (4)	11 (14)
Kruskal-Wallis rank test		p= 0.4532	p= 0.0727	p= 0.0192	p= 0.0100	p= 0.0132
*CARIES EXPERIENCE (DMFS)*						
0	205	4 (4)	3 (5)	3 (5)	2 (4)	13 (14)
1-2	110	3.5 (3)	2 (5)	3 (6)	1.5 (5)	11 (14)
3-5	131	4 (2)	3 (5)	3 (5)	2 (5)	12 (16)
>=6	113	4 (3)	3 (4)	4 (7)	1 (4)	13 (14)
Kruskal-Wallis rank test		p= 0.2516	p= 0.6599	p= 0.3348	p= 0.9985	p= 0.6951
*HAD ALREADY UNDERGONE ORTHODONTIC TREATMENT*						
no	444	4 (3)	2.5 (4)	3 (5)	1 (4)	12 (13)
yes	115	4 (4)	4 (5)	3 (5)	3 (5)	17 (16)
Wilcoxon rank-sum test		p= 0.0081	p< 0.0001	p= 0.4071	p< 0.0001	p< 0.0001

## Discussion

Studies assessing the impact of oral disorders on quality of life have been conducted since the 1980’s. Originally published in English, the CPQ_11-14_ is the most commonly used instrument to evaluate children/adolescent self-perception about oral health. This study aimed to translate, adapt, and validate an Italian version of the original English questionnaire in order to assess the impact of oral disorders on overall quality of life in Italian children/adolescents.

There was a good overall response rate to the items on the questionnaire (98%): functional limitations was the section in which the respondents left the highest number (8) of blanks. As there did not seem to be any specific pattern in the non-responses, we concluded that the low number of unanswered items would not affect our analyses.

A good internal consistency was found: Cronbach’s alpha resulted 0.88 for the total score while it was 0.85 for both the social well-being and functional limitations domains. In their original study, Jokovic and coll. [11] showed Cronbach’s alpha ranging from 0.64 to 0.91: they also reported high levels of reliability (by means of test-retest) indicating that the questionnaire is reliable and stable over time periods, achieving a very high agreement level by intra-class correlation coefficient (0.9) on children with different oral health conditions. Other validation studies also reported a good internal consistency [12-19].

Although statistically significant, the correlation coefficients in the construct validity analysis were low, as were those reported by other studies, which in any case considered the questionnaire valid for the populations (both English and non-English) being assessed [11,15,17-19]. The construct validity of our survey was in any case as high as that reported by those works.

Except for the functional limitation domain and global rating of oral health (p = 0.1573) relationship, the correlations between the respondents’ global rating of oral health and well-being and the total and sub-scale scores were all highly significant. These findings were similar to those outlined by Jokovic et al. [11]. It can by hypothesized that the respondents were unable to make any connection between theoretical aspects concerning their oral health status and functional limitations. Different social classes seemed to be associated to distinctive response patterns in the total score and in the emotional and social well-being sub-scores. Socio-economic status is, in fact, a well established predictor of oral-health-related quality of life [22,27,28]. These findings confirm the need to consider SES when studying oral health status and planning health strategies.

CPQ_11-14_ scores identified clear differences between the two genders and in the groups which had or did not have orthodontic treatment. Orthodontic treatment could modify one's oral health perception. Researchers have reported varied effects of orthodontic treatment on HRQoL [29], showing conflicting findings [19,30,31]. We notwithstanding did not excluded adolescents with such a treatment because this work (a part of a research project) aimed to test the validity of the Italian adaptation of the questionnaire: the subgroup of adolescents treated improved the performance of this study giving a further insight in discriminant validity testing.

Differences between genders may, nevertheless, be due to the commonly reported “sex effect” on perception of health status [12]. Respondents with or without caries experience did not produce a significant gradient in the CPQ_11-14_ scores. It can be hypothesized that carious teeth did not affect the aspects of oral health and well-being considered in the questionnaire [8]. Other authors recently found a relationship between DMFS and OHRQoL via an indirect effect [32], however having a little effect [33]. As a general rule, we need to remember that outcomes on any health-related questionnaire may be affected by clinical as well as unforeseeable cultural, social, environmental, sexual, or individual factors. The present study presents some limitations. For one, the sampling procedure was restricted to only one geographical area and its results may not reflect the rest of the population.

The study may, moreover, present a selection bias due to differences between the characteristics of the children/adolescents participating in the study and those of the non-participants. It is, nonetheless, true that 782 parents (almost 71% of the original number approached) answered the self-report social status questionnaires although only 561 returned signed consent forms permitting their children/adolescents to participate. We were thus able to verify that the distribution of social positions was not significantly different in the participants and non-participants. The fact that the classes had been randomly selected certainly favoured the population representativity of our sample and it is probable that any selection bias only minimally affected the results.

We didn’t perform a test-retest reliability assessment of the questionnaire: although due to respondent burden it could represent a limitation of the study. Kok et al. [34] did not undertake any re-testing because of the high levels of reliability previously reported and in view of the fact that individuals tend to adapt to or become used to their (health) conditions over time.

We worked on the “long version” of the CPQ_11-14_ although it has been proposed and validated short forms of the same tool [28]. Given the aim of the present study we choose to adopt the long form in order to test the validity of the Italian version of the original questionnaire. Moreover we found an excellent response rate (98%) avoiding the risk of total non-response with a very low number of blank items.

## Conclusions

In conclusion, the newly translated Italian version of the CPQ_11-14_ utilized in this study has been validated and showed good internal consistency and construct and discriminant validity and seems to be a valid instrument for measuring oral health-related quality of life in Italian children/adolescents.

These findings confirm that a relationship between oral health status and OHRQoL in children can be explored focusing our knowledge on the patient rather than just the disease.

## Competing interests

This project was supported with a grant awarded by way of donation from a private company (Leone Spa, an Italian manufacturer of orthodontic products located in Sesto Fiorentino) which had no say as to how the study was to be carried out. All authors declare that they have no conflicting interests.

## Authors’ contributions

AO, RF, LB, AB, ES conceived and designed the original protocol. All the authors were involved in modifying the protocol and approving the final draft. AB and RF examined the children/adolescents and collected data. AO and RF coordinated all of the activities throughout the various stages of the project. Data entry was carried out by AB and RF. AO and LB cleaned the data and performed the data analyses and the others made suggestions. AO wrote the first draft of the manuscript with RF and AB. All the authors contributed to preparing the subsequent and approved the final drafts.

## Pre-publication history

The pre-publication history for this paper can be accessed here:

http://www.biomedcentral.com/1472-6831/13/55/prepub

## References

[B1] Centers for Disease Control and Prevention Measuring healthy days: Population assessment of health-related quality of life 2000 Atlanta, Georgia: Centers for Disease Control and Prevention

[B2] Cohen LK The emerging field of oral health-related quality of life outcomes research. In Measuring oral health and quality of life. Edited by Slade GD. University of North Carolina 1997 Chapel Hill: University of North Carolina, Dental Ecology

[B3] Cushing AM Sheiham A Maizels J Developing socio-dental indicators—the social impact of dental disease Community Dent Health 1986 3 3 17 3516317

[B4] Atchison KA Dolan TA Development of the Geriatric Oral Health Assessment Index J Dent Educ 1990 54 680 687 2229624

[B5] Locker D Miller AM Subjectively reported oral health status in an adult population Community Dent Oral Epidemiol 1994 22 425 430 10.1111/j.1600-0528.1994.tb00791.x 7882657

[B6] Slade GD Spencer AJ Development and evaluation of the Oral Health Impact Profile Community Dent Health 1994 11 3 11 8193981

[B7] Leão A Sheiham A The development of a socio-dental measure of dental impacts on daily living Community Dent Health 1996 13 22 26 8634892

[B8] Barbosa TS Gaviao MBD Oral health-related quality of life in children: part II. Effects of clinical oral health status. A systematic review Int J Dent Hyg 2008 6 100 106 10.1111/j.1601-5037.2008.00293.x 18412721

[B9] Gherunpong S Tsakos G Sheiham A Developing and evaluating an oral health-related quality of life index for children; the CHILD-OIDP Community Dent Health 2004 21 161 169 15228206

[B10] Broder HL Wilson-Genderson M Reliability and convergent and discriminant validity of the Child Oral Health Impact Profile (COHIP Child’s Version) Community Dent Oral Epidemiol 2007 35 Suppl. 1 20 31 17615047 10.1111/j.1600-0528.2007.0002.x

[B11] Jokovic A Locker D Stephens M Kenny D Tompson B Guyatt G Validity and reliability of a questionnaire for measuring child oralhealth-related quality of life J Dent Res 2002 81 459 463 10.1177/154405910208100705 12161456

[B12] O'Brien K Wright JL Conboy F Macfarlane T Mandall N The child perception questionnaire is valid for malocclusions in the United Kingdom Am J Orthod Dentofacial Orthop 2006 129 4 536 540 10.1016/j.ajodo.2004.10.014 16627180

[B13] Locker D Jokovic A Thomson WM Foster Page LA Validation of the Child Perceptions Questionnaire (CPQ11-14) J Dent Res 2005 84 649 652 10.1177/154405910508400713 15972595

[B14] Do LG Spencer AJ Evaluation of oral health-related quality of life questionnaires in a general child population Community Dental Health 2008 25 205 210 19149296

[B15] Brown A Al-Khayal Z Validity and reliability of the Arabic translation of the child oral health related quality of life questionnaire (CPQ11-14) in Saudi Arabia Int J Paediatr Dent 2006 16 405 411 10.1111/j.1365-263X.2006.00775.x 17014538

[B16] Robinson PG Nalweyiso N Busingye J Whitworth J Subjective impacts of dental caries and fluorosis in rural Ugandan children Community Dent Health 2005 22 4 231 236 16379161

[B17] Goursand D Paiva SM Zarzar PM Ramos-Jorge ML Cornacchia GM Pordeus IA Allison PJ Cross-cultural adaptation of the Child Perceptions Questionnaire 11–14 (CPQ11-14) for the Brazilian Portuguese language Health Qual Life Outcomes 2008 14 2 18194552 10.1186/1477-7525-6-2 PMC2246108

[B18] McGrath C Pang HN Lo EC King NM Hägg U Samman N Translation and evaluation of a Chinese version of the Child Oral Health-related Quality of Life measure Int J Paediatr Dent 2008 18 267 274 18554335 10.1111/j.1365-263X.2007.00877.x

[B19] Wogelius P Gjørup H Haubek D Lopez R Poulsen S Development of Danish version of child oral-health-related quality of life questionnaires (CPQ8-10 and CPQ11-14) BMC Oral Health 2009 9 11 10.1186/1472-6831-9-11 10.1186/1472-6831-9-11 19383176 PMC2679003

[B20] O'Brien C Benson PE Marshman Z Evaluation of a quality of life measure for children with malocclusion J Orthod 2007 34 185 193 10.1179/146531207225022185 17761802

[B21] Feu D de Oliveira BH de Oliveira Almeida MA Kiyak HA Miguel JA Oral health-related quality of life and orthodontic treatment seeking Am J Orthod Dentofacial Orthop 2010 138 152 159 10.1016/j.ajodo.2008.09.033 20691356

[B22] Caiazzo A Cardano M Cois E Costa G Marinacci C Spadea T Vannoni F Venturini L Inequalities in health in Italy Epidemiol Prev 2004 28 3 1 161 15537046

[B23] Behling O Law K Lewis-Beck MS Translating questionnaires and other research instruments Problems and Solutions Quantitative Applications in the Social Sciences 2000 07–131 Thousand Oaks: Sage 1 70

[B24] Meadows K Bentzen N Touw-Otten F Hutchinson A, Bentzen N, König-Zahn C Cross-cultural issues: an outline of the important principles in establishing cross-cultural validity in health outcome assessment Cross Cultural Health Outcome Assessment; a user's guide 1996 Ruiner NL: ERGHO; European Research Group on Health Outcomes 34 40

[B25] Herdman M Fox-Rushby J Badia X A model of equivalence in the cultural adaptation of HRQoL instruments: the universalist approach Qual Life Res 1998 7 4 323 335 10.1023/A:1008846618880 9610216

[B26] Streiner DL Norman GR Health measurement scale: practical guide to their development and use 2003 New York: Oxford

[B27] Locker D Disparities in oral health-related quality of life in a population of Canadian children Community Dent Oral Epidemiol 2007 35 348 356 10.1111/j.1600-0528.2006.00323.x 17822483

[B28] Watt R Sheiham A Inequalities in oral health: a review of the evidence and recommendations for action Br Dent J 1999 187 1 6 12 10452185 10.1038/sj.bdj.4800191

[B29] Taylor KR Kiyak A Huang GJ Greenlee GM Jolley CJ King GJ Effects of malocclusion and its treatment on the quality of life of adolescents Am J Orthod Dentofacial Orthop 2009 136 3 382 92 10.1016/j.ajodo.2008.04.022 19732673

[B30] Agou S Locker D Muirhead V Tompson B Streiner DL Does psychological well-being influence oral-health-related quality of life reports in children receiving orthodontic treatment? Am J Orthod Dentofacial Orthop 2011 139 3 369 77 10.1016/j.ajodo.2009.05.034 21392693

[B31] Aa C Ferreira MC Serra-Negra JM Pordeus IA Paiva SM Impact of wearing fixed orthodontic appliances on oral health-related quality of life among Brazilian children J Orthod 2011 38 4 275 81 10.1179/14653121141632 22156183

[B32] Foster Page LA Thomson WM Ukra A Baker SR Clinical status in adolescents: is its impact on oral health-related quality of life influenced by psychological characteristics? Eur J Oral Sci 2013 121 3 Pt 1 182 7 23659241 10.1111/eos.12034

[B33] Foster Page LA Thomson WM Caries prevalence, severity, and 3-year increment, and their impact upon New Zealand adolescents’oral-health-related quality of life J Public Health Dent 2012 72 4 287 94 10.1111/j.1752-7325.2012.00336.x 22506615

[B34] Kok YV Mageson P Harradine NW Sprod AJ Comparing a quality of life measure and the Aesthetic Component of the Index of Orthodontic Treatment Need (IOTN) in assessing orthodontic treatment need and concern J Orthod 2004 31 4 312 8 10.1179/146531204225020625 15608346

